# The effect of acupuncture combined with hyperbaric oxygenation compared with hyperbaric oxygenation alone for patients with traumatic brain injury: a systematic review and meta-analysis

**DOI:** 10.3389/fneur.2025.1538740

**Published:** 2025-05-02

**Authors:** Geng Li, Binyang Wang, Shuochen Fan, Shuangli Liu, Lu Shao, Chuanxiong Li, Yongjiang Fang, Jianfeng Li, Meihua Qiu, Yuping Zhang, Lei Pan

**Affiliations:** ^1^The Second Clinical Medical College, Yunnan University of Traditional Chinese Medicine, Kunming, China; ^2^Kunming Medical University, Kunming Medical University, Kunming, China; ^3^Department of Rehabilitation Medicine, Affiliated Hospital of Yunnan University, Kunming, China; ^4^The Third Affiliated Hospital, Yunnan University of Chinese Medicine, Kunming, China; ^5^The Second Affiliated Hospital, Yunnan University of Traditional Chinese Medicine, Kunming, China; ^6^Department of Rehabilitation Medicine, Yunnan Integrated Hospital of Traditional Chinese and Western Medicine, Kunming, China

**Keywords:** acupuncture, hyperbaric oxygenation, traumatic brain injury, Glasgow Coma Scale, systematic review, meta-analysis

## Abstract

**Objective:**

This study aimed to evaluate the effects of acupuncture combined with hyperbaric oxygenation (HBO) compared with HBO alone in improving the disturbance of consciousness (DOC) of people with traumatic brain injury (TBI).

**Methods:**

The Preferred Reporting Items for Systematic Reviews and Meta-Analyses (PRISMA) guidelines were followed in this study. In accordance with the preestablished search strategy, all the literature was obtained from eight online databases. Following the stringent application of inclusion and exclusion criteria, two researchers conducted an independent extraction of valid data from eligible randomized controlled trials (RCTs). The risk of bias in each study was assessed using the Cochrane Risk of Bias 2.0 tool. The meta-analysis was conducted utilizing the RevMan software. Adverse events were determined based on data from each study assessing the safety of acupuncture treatment.

**Results:**

A total of 11 RCTs with 896 participants were included in the analysis. Overall, the methodological quality of the RCTs encompassed within this meta-analysis was below standard. The pooled data demonstrated that acupuncture treatment combined with HBO was significantly superior to HBO alone, based on the Glasgow Coma Scale (GCS) scores [mean difference (MD) = 2.13, 95% confidence interval (CI): 1.64–2.62, *p* < 0.00001]. We also found that electroacupuncture (EA) combined with HBO improved GCS scores more than HBO alone in TBI patients (MD = 2.15, 95% CI: 1.95–2.36). The early intervention (MD = 3.09, 95% CI, 2.66–3.52) demonstrated significantly more significant improvement in GCS scores following combination therapy compared with the late intervention group (MD = 1.86, 95% CI, 1.47–2.25). Furthermore, compared with HBO alone, acupuncture combined with HBO significantly improved patients’ consciousness rate (CR) (RR = 2.26, 95% CI: 1.48–3.46). Statistical analysis also revealed that acupuncture combined with HBO improved the effective rate (ER) (RR = 1.47, 95% CI: 1.27–2.69). Additionally, no studies reported any significant adverse events.

**Conclusion:**

Compared with HBO alone, acupuncture combined with HBO has a more substantial positive effect on GCS scores, AR, and ER in patients with TBI. However, given the limited availability of high-quality evidence and the dearth of RCTs in this area, the conclusions drawn herein warrant validation through additional research endeavours.

**Clinical trial registration:**

https://www.crd.york.ac.uk/PROSPERO/view/CRD42024576067, CRD42024576067.

## 1 Introduction

Traumatic brain injury (TBI) is a neurological disease caused mainly by violent injury to the head. This trauma can damage the brain and lead to a series of symptoms, such as disturbance of consciousness (DOC), cognitive impairment, and psychological and physiological dysfunctions (1). A document from the Lancet Neurology Commission states that by 2030, TBI is estimated to remain one of the top three causes of injury-related death and disability, with 50–60 million people affected by the disease each year worldwide, with 5.48 million cases classified as severe (2).

Currently, clinical treatments for TBI include hyperbaric oxygenation (HBO) (3), medication, brain stimulation (4), and traditional rehabilitation therapy (5). In addition, aural-musical therapy (6), robot-assisted therapy devices (7), and brain-computer interfaces (8) are garnering increasing attention and being incorporated into research and clinical practices. Although significant progress has been made over the past two decades in stimulating the resurgence of awareness in TBI patients, the available therapeutic modalities remain limited (9). Developing more efficacious, simplified, and comprehensive restorative and therapeutic approaches is vital for optimizing TBI treatment. Acupuncture is a commonly used technique in China for treating neurological disorders (10). Studies have shown its neuroprotective effects in traumatic brain injury (TBI), including improved consciousness, alleviated cognitive dysfunction, and reduced mortality rates (11). HBO is a crucial component in the comprehensive treatment of cranial brain injuries, offering a multitude of significant benefits (12). These include enhancing blood oxygen levels to combat hypoxia, alleviating cerebral edema and reducing intracranial pressure, stimulating the central reticular system to improve consciousness, fostering blood circulation and neural repair, exerting anti-inflammatory and immune-modulating effects, and hastening the clearance of lesions and the absorption of hematomas (13, 14).

There has been no known synthesis of evidence comparing acupuncture combined with HBO and HBO alone. Therefore, the core aim of this systematic review and meta-analysis is to determine whether acupuncture combined with HBO improves TBI symptoms more than HBO alone.

## 2 Materials and methods

This study was conducted according to the Preferred Reporting Items for Systematic Reviews and Meta-Analyses (PRISMA) guidelines (15). This study was registered on the Prospective Register of Systematic Reviews (PROSPERO) on August 16^th^, 2024, with the registration number CRD42024576067.

### 2.1 Search strategy

In this study, the following databases were searched: Medline, Embase, the Cochrane Central Register of Controlled Trials (CENTRAL), Web of Science (SCI), Chinese Biomedical Database (CBM), Chinese National Knowledge Infrastructure (CNKI), Wan Fang Data Knowledge Service Platform, and VIP Journal Integration Platform (VIP). All evidence published prior to September 7^th^, 2024, was included. Reference lists from studies identified through the Chinese Clinical Trial Register and ClinicalTrials.gov were also scrutinized, and any additional studies identified through this process were included. The MeSH function was utilized to locate studies using synonyms of the keywords entered in this search process. The keywords included “Acupuncture,” “Hyperbaric oxygenation,” and “Traumatic brain injury.” The specific search strategies employed have been detailed in the Supplementary material.

### 2.2 Selection criteria

Below are the criteria guiding this review:

(1) Subjects must have a diagnosis of TBI.
(2) The condition must be verified via magnetic resonance imaging (MRI) or computed tomography (CT).
(3) In the treatment group, acupuncture combined with HBO was used, while HBO was used as the primary treatment in the control group. Both groups also received the same basic treatments.
(4) The study type was an RCT.

### 2.3 Exclusion criteria

Studies that were excluded based on the following criteria:

(1) The study was a duplication of a previous study.
(2) The study compared different acupuncture therapies without the inclusion of HBO.
(3) The full text was not available.

### 2.4 Study selection and data extraction

Two researchers (BY-W and SC-F) conducted independent screenings of the studies, excluding duplicates and those deemed irrelevant. Each reviewer screened articles to ensure they met the inclusion criteria. Data extraction from eligible studies was conducted and verified, with discrepancies resolved through consultation. When consensus was not reached, a third researcher (SL-L) was invited to provide a decisive opinion. All excluded literature was systematically documented. Data extraction was conducted independently by CX-L and YJ-F, with validation performed by researcher BY-W. Extracted data included first author, publication year, number of participants, age, treatment duration, diagnostic criteria, types of outcomes, side effects and adverse events, and other relevant information. The results were recorded in an Excel spreadsheet.

### 2.5 Study quality assessment

The quality of individual studies was assessed using the Cochrane Risk of Bias 2.0 tool (16). The following six items were extracted from each of the RCTs: (a) randomization process; (b) deviations from intended interventions; (c) missing outcome data; (d) measurement of the outcome; (e) selection of the reported results; (f) overall findings. Studies employing suitable methodologies and providing transparent descriptions were deemed to exhibit a low bias risk. In contrast, those lacking such methodological rigor and clarity in the description were categorized as having a high bias risk. SL-L and LS performed a bilateral evaluation of these factors, with a third researcher (BY-W) providing an objective opinion and helping to resolve any ensuing disputes.

### 2.6 Types of outcomes

The primary outcome measure was the Glasgow Coma Scale (GCS) score. The GCS was developed more than 50 years ago to measure the “depth and duration of impaired consciousness” of patients with TBI (17). Five decades of research support the efficacy of this tool in assessing and describing patients with neurological impairment (18).

One secondary outcome was the Consciousness Rate (CR), which included patients who reached a minimally conscious state (MCS) and those who emerged from MCS, as assessed by the Coma Recovery Scale-Revised (CRS-R), and were judged to be conscious. Another secondary outcome was the Effective Rate (ER), calculated as the sum of effective and improved cases divided by the total number of cases. Treatment effectiveness was determined if clinical symptoms completely disappeared, consciousness was clear, speech was fluent, and no assistance was needed in daily life. Clinical symptoms were considered improved if there was a significant improvement in DOC and daily life required only mild assistance. Treatment was deemed ineffective if there was no or only slight improvement in clinical symptoms or consciousness, and full assistance was required in daily life.

### 2.7 Statistical analysis

The RevMan 5.4 software package was used to perform the statistical analyses. For categorical variables, the risk ratio (RR) and its corresponding 95% confidence interval (CI) were applied. For continuous variables, the mean difference (MD) was chosen as the metric, with a 95% CI. Statistical significance was set at *p* < 0.05. The Q test and I^2^ statistics were used to assess heterogeneity between the included studies, and the effect model was selected based on these assessments. Statistically significant heterogeneity among the included studies was indicated by *p* < 0.1 and I^2^ > 50%. Consequently, a random-effects model was used to determine the effect size for each outcome. When *p* ≥ 0.1 and I^2^ < 50%, the heterogeneity among the studies was considered acceptable, leading to the use of a fixed-effects model for data synthesis in these cases. Combined results were assessed using the Z test, with *p* < 0.05 indicating a statistically significant difference. Publication bias within the included studies was assessed using funnel plots and Egger’s regression test. All forest plots were oriented with effect sizes calculated as experimental group minus control group MD or RR for the experimental group, with “Favors acupuncture + HBO” clearly labelled on the right side to prevent directional misinterpretation.

## 3 Results

### 3.1 Studies retrieved

**Figure 1:**
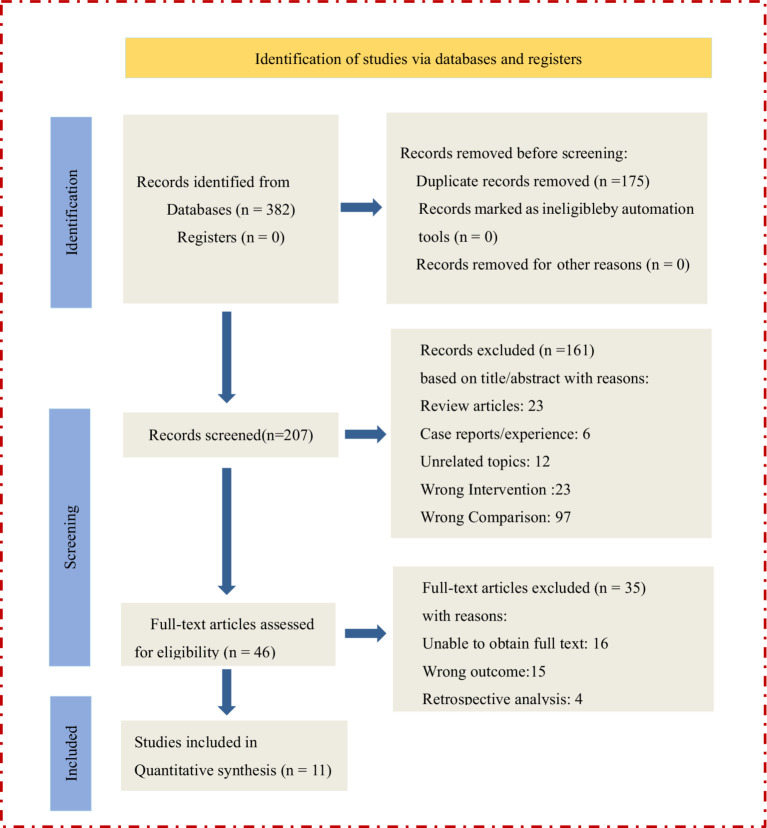
Flowchart of the study selection process.

Figure 1 presents a detailed flow chart of the study selection process according to the PRISMA guidelines. First, we removed 175 duplicate articles, and the titles and abstracts of the remaining 207 articles were screened. After screening, 51 full texts were reviewed. A total of 11 RCTs were included in the meta-analysis (19–29).

### 3.2 Characteristics of the study participants

A total of 896 participants were included, 448 in the experimental group and 448 in the control group. The study sample sizes ranged from 28 to 158 participants. There were more males than females in the study population. No significant difference in the mean age was found between the two groups (*p* > 0.05). Demographic details of the 11 included RCTs are summarized in Table 1.

**Table 1:** Characteristic of included studies.

Study	No. of patients (O/A)	Age: mean ± SD or range (years)	Period of Treatment	Disease duration (day)	Initial GCS Score	Side effects and adverse events	Type of outcomes
Li et al. (19)	I: 40/40 C: 40/40	I: 49.45 ± 10.12 C: 50.05 ± 10.26	1 month	x̄: 28.37 ± 6.76	I: 5.95 ± 1.42 C: 5.88 ± 1.40	NR	GCS, CR, GOS, MBP, NSE, S100β
Ying et al. (20)	I: 30/30 C: 29/30	I: 50.47 ± 6.18 C: 48.47 ± 7.67	1 month	I: 40.76 ± 8.96 C: 39.55 ± 7.59	I: 6.53 ± 1.83 C: 6.44 ± 1.55	NR	GCS, ER
Bao et al. (21)	I: 48/50 C: 50/50	I: 44.00 ± 12.00 C: 45.00 ± 15.00	1 month	I:30.30 ± 10.20 C:30.6 ± 16.80	I: 5.92 ± 1.31 C: 5.86 ± 1.39	NR	GCS, CR, CRS-R
Zhao et al. (22)	I: 79/79 C: 79/79	I: 43.62 ± 8.15 C: 42.70 ± 8.57	20 days	^−^ x: 0.51 + 0.18	I: 5.27 ± 1.14 C: 5.33 ± 1.32	NR	GCS, MMSE, GQOLI-74, NIHSS, Barthel
Xiang et al. (23)	I: 14/14 C: 14/14	x̄: 48.12	10 days a course of treatment	x̄: 28.26	I: 5.22 ± 1.63 C: 5.23 ± 1.53	Pain, Fever	GCS, GOS
Zheng and Zhang (24)	I: 18/18 C: 18/18	I: 44.32 ± 5.91 C: 43.03 ± 5.60	3 weeks	I: 31.32 ± 5.91 C: 32.65 ± 5.17	I: 9.52 ± 1.67 C: 9.43 ± 1.57	NR	GCS, ER, CRS-R
Zhao et al. (25)	I: 30/32 C: 32/32	x̄:15.00–60.00	1 month	≤60.00	I: 5.76 ± 1.06 C: 5.32 ± 1.12	NR	GCS
Wang et al. (26)	I: 73/73 C: 73/73	I: 41.4 ± 2.90 C: 43.9 ± 2.60	2 months	I: 45.00 ± 12.00 C: 42.00 ± 9.00	I: 5.28 ± 0.87 C: 5.52 ± 0.93	NR	GCS, ER, CR
Che et al. (27)	I: 45/45 C: 45/45	I: 43.22 ± 9.13 C: 41.14 ± 8.33	10 days a course of treatment	I: 15.21 ± 6.27 C: 14.93 ± 5.94	I: 7.08 ± 1.75 C: 7.22 ± 1.62	NR	GCS, ER, NIHSS, QLQ-C30
Chen et al. (28)	I: 39/39 C: 38/38	I: 31.47 ± 3.88 C: 31.52 ± 3.82	1 month	NR	<8.00	NR	ER
Lu et al. (29)	I: 32/32 C: 30/30	x̄: 34.44	2 months	x̄: 40.48	NR	NR	CR, EEG, BAEP, DA, NE

GOS, Glasgow Outcome Scale; MBP, Myelin basic protein; NSE, Neuron specific enolase; MMSE, Mini Mental State Examination; GQOLI-74, Generic Quality of Life Inventory-74; NIHSS, National Institutes of Health Stroke Scale; QLQ-C30, Quality of Life Questionnaire Core 30; EEG, Electroencephalography; BAEP, Brainstem auditory evoked potentials; DA, Dopamine; NE, Norepinephrine. NR, not reported.

### 3.3 Description of interventions

Electroacupuncture (EA) was used most frequently (54.5%), followed by hand acupuncture (MA) (36.4%) and warm acupuncture (WA) (9%). All included studies utilized HBO as a control measure. The needle retention time was set at 30 min in 10 out of 11 studies. The frequency of treatment sessions varied between 5 and 7 times weekly, with an average frequency of 5 sessions (*n* = 5). The treatment periods in these studies ranged from 10 days to 8 weeks, with a 4-week treatment period being optimal (*n* = 5). Two studies (24, 27) did not report treatment periods, and the length of time was determined on the basis of the patients’ disease progression. The following points and frequencies were noted in the included studies: Shui-gou (63.6%), Nei-guan (54.5%), Bai-hui (54.5%), San-yin-jiao (45.5%), Yin-tang (36.4%), He-gu (36.4%), and Tai-chong (27.3%). These acupoints were employed with the most significant frequency. Table 2 depicts the characteristics of the interventions in the included studies.

**Table 2:** Specifics of the interventions within the reviewed studies.

Study	Acupuncture methods		Treatment duration		
	Style	Acupoint selection	Frequency	Retained	Course
Li et al. (19)	MA	Bai-hui (DU20), Shui-gou (DU26), Nei-guan (PC6), He-gu (LI4), Tai-chong (LR3)	5 times/w	30 min	4 weeks
Ying et al. (20)	MA	Shui-gou (DU26), Nei-guan (PC6), San-yin-jiao (SP6)	7 times/w	30 min	4 weeks
Bao et al. (21)	MA	Shui-gou (DU26), Nei-guan (PC6), San-yin-jiao (SP6), Chi-ze (LU5), Wei-zhong (BL40), He-gu (LI4), Tai-chong (LR3)	5 times/w	30 min	4 weeks
Zhao et al. (22)	WA	Yong-quan (KI1), Ji-quan (HT1), Wei-zhong (BL40)	5 times/w	30 min	4 weeks
Xiang et al. (23)	EA	Shui-gou (DU26), Nei-guan (PC6), San-yin-jiao (SP6), Bai-hui (DU20), Ren-ying (ST9), Hou-xi (SI3)	NR	18–24 min	NR
Zheng and Zhang (24)	MA	Bai-hui (DU20), Si-shen-cong (EX-HN1), Shen-ting (DU24), Tou-wei (ST8), Ying-tang (DU29), Shui-gou (DU26), Feng-fu (DU16), Ya-men (DU15), Nei-guan (PC6), San-yin-jiao (SP6)	6 times/w	30 min	3 weeks
Zhao et al. (25)	EA	Bai-hui (DU20), Ying-tang (DU29), Shui-gou (DU26), Feng-chi (GB20), San-yin-jiao (SP6)	5 times/w	30 min	4 weeks
Wang et al. (26)	EA	Bai-hui (DU20), Shui-gou (DU26), Nei-guan (PC6), He-gu (LI4), Tai-chong (LR3), Si-shen-cong (EX-HN1), Zu-san-li (ST36), San-yin-jiao (SP6), Ren-ying (ST9), Hou-xi (SI3)	NR	30 min	8 weeks
Che et al. (27)	EA	Qu-ze (PC3), Xi-men (PC4), Jian-shi (PC5), Nei-guan (PC6), Da-ling (PC7)	5 times/w	30 min	NR
Chen et al. (28)	EA	Bai-hui (DU20), He-gu (LI4), Ying-tang (DU29), Tou-wei (ST8), Feng-fu (DU16), Feng-chi (GB20), Nao-hu (DU17), Tong-Tian (BL7), Yin-men (BL37), Tian-zhu (BL10), Wai-guan (SJ5)	7 times/w	30 min	10 days
Lu et al. (29)	EA	Bai-hui (DU20), Shui-gou (DU26)	NR	30 min	8 weeks

MA, manual acupuncture; WA, warm acupuncture; EA, Electroacupuncture.

### 3.4 Quality assessment results

**Figure 2:**
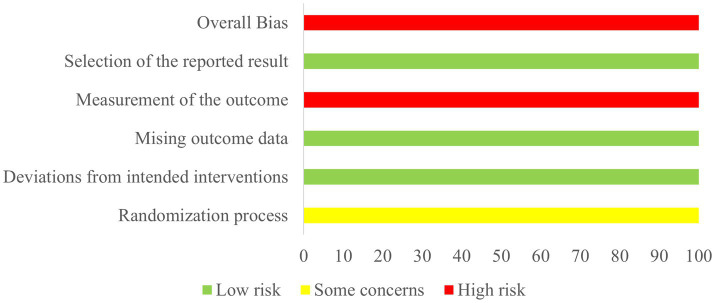
Risk of bias summary.

**Figure 3:**
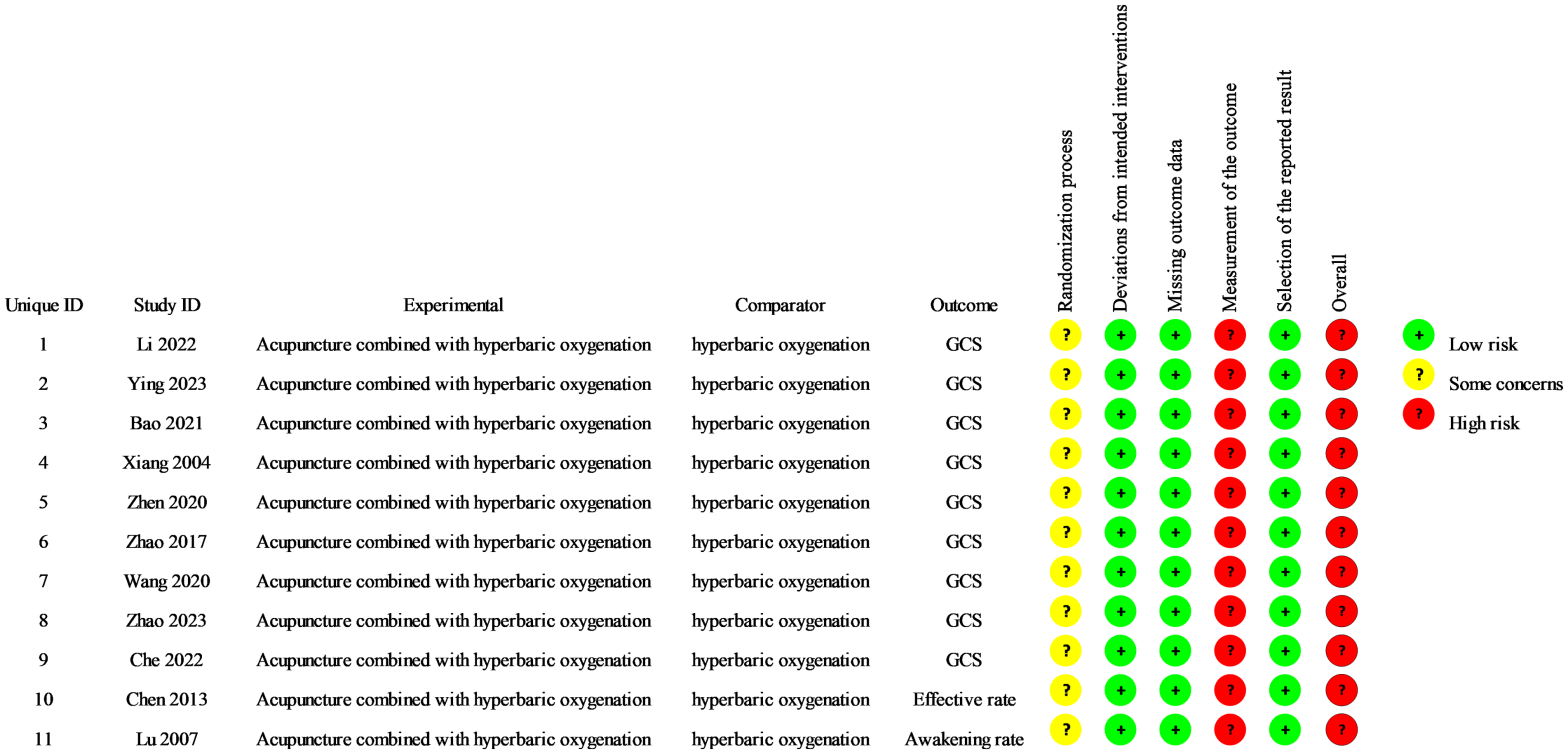
Risk of bias graph.

The methodological rigor of the encompassed studies was consistently found to be subpar. All included trials reported on randomization processes. Three studies (22, 26, 28) only mentioned the word ‘randomization’ without detailing the method used. None of the included studies detailed the methods of group allocation, leading to an assessment of moderate bias risk. None of the included studies reported blinding methods and were classified as exhibiting a high risk of bias. No other methodological deviations were found in the included studies (Figures 2, 3).

### 3.5 Meta-analysis

#### 3.5.1 Glasgow coma scale

**Figure 4:**
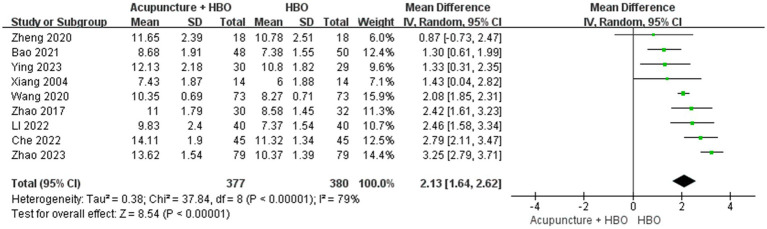
Forest plot of effects of acupuncture combined with HBO versus HBO alone on GCS score (Random effect model). MD > 0 indicates that the acupuncture combined with HBO group demonstrated higher GCS scores.

Nine studies (19–27) adopted the GCS score to evaluate patients’ level of consciousness impairment. Due to significant heterogeneity (*p* < 0.00001, I^2^ = 79%), a random-effects model was employed. Significantly higher Glasgow Coma Scale (GCS) scores were demonstrated in patients receiving combined acupuncture and HBO therapy compared to those undergoing HBO treatment alone. (MD = 2.13, 95% CI: 1.64–2.62, *p* < 0.00001) (Figure 4).

**Figure 5:**
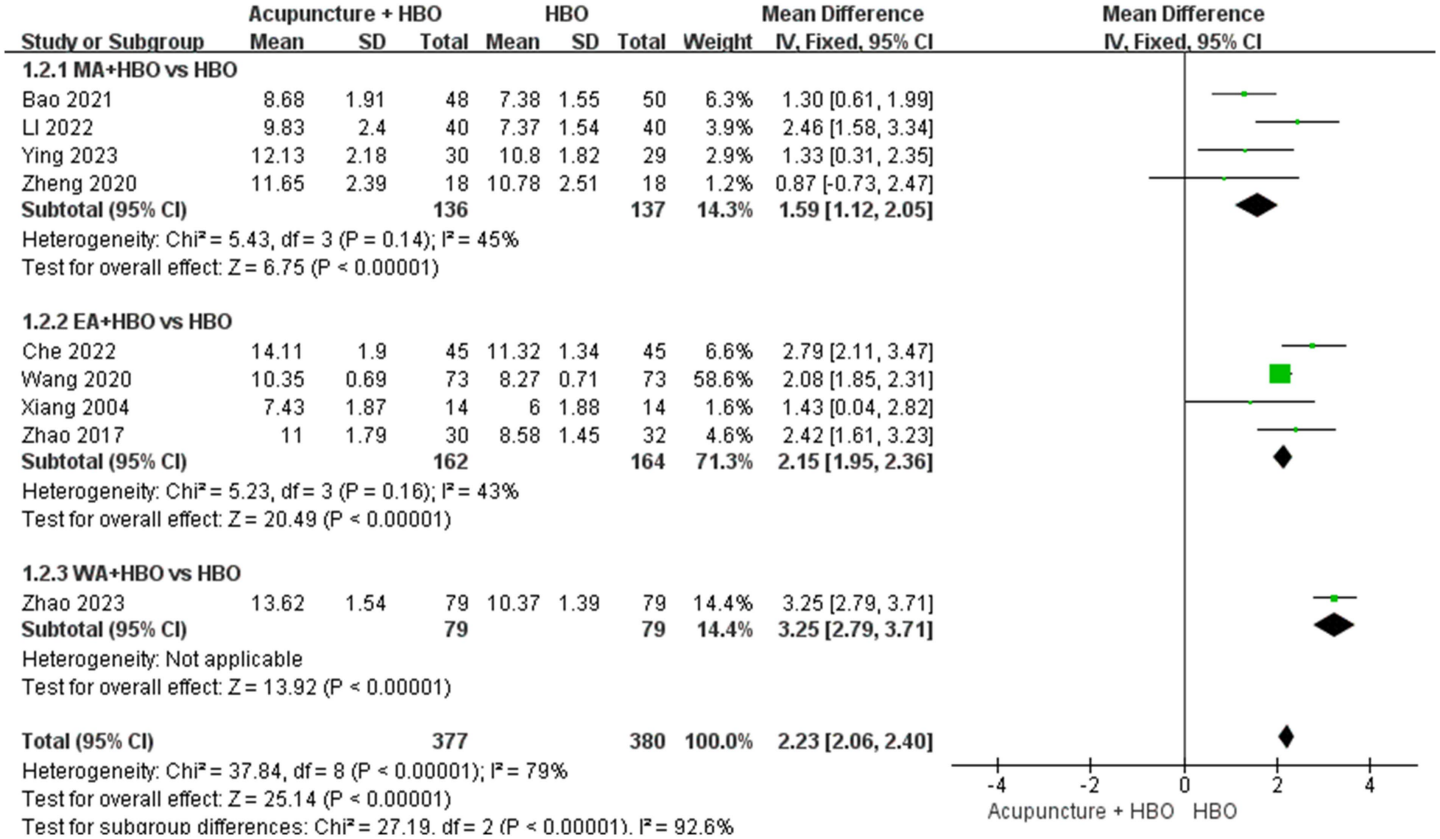
Forest plot of effects of acupuncture combined with HBO versus HBO alone on GCS score (Subgroup by the different acupuncture style). MD > 0 indicates that the acupuncture combined with HBO group demonstrated higher GCS scores.

The stratified analyses were performed by acupuncture modality: manual acupuncture (MA), electroacupuncture (EA), and warm acupuncture (WA). Subgroup analysis revealed statistically significant between-subgroup heterogeneity (*p* < 0.00001, I^2^ = 92.6%). The EA subgroup demonstrated a superior improvement in GCS scores (MD = 2.15, 95% CI: 1.95–2.36) with low heterogeneity (I^2^ = 43%, *p* = 0.16), compared with the MA subgroup (MD = 1.59, 95% CI: 1.12–2.05, *p* < 0.00001, I^2^ = 45%, *p* = 0.14). The WA subgroup was excluded from the pooled analysis due to insufficient sample size (*n* = 1) (Figure 5).

**Figure 6:**
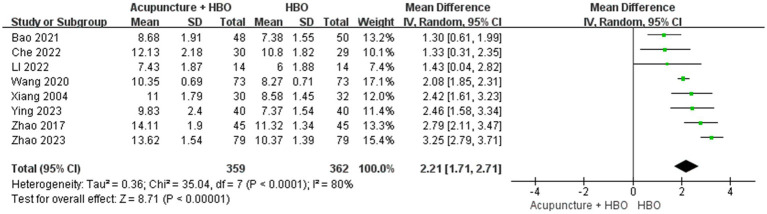
Forest plot of effects of acupuncture combined with HBO versus HBO alone on GCS score (Sensitivity analysis of TBI classification). MD > 0 indicates that the acupuncture combined with HBO group demonstrated higher GCS scores.

**Figure 7:**
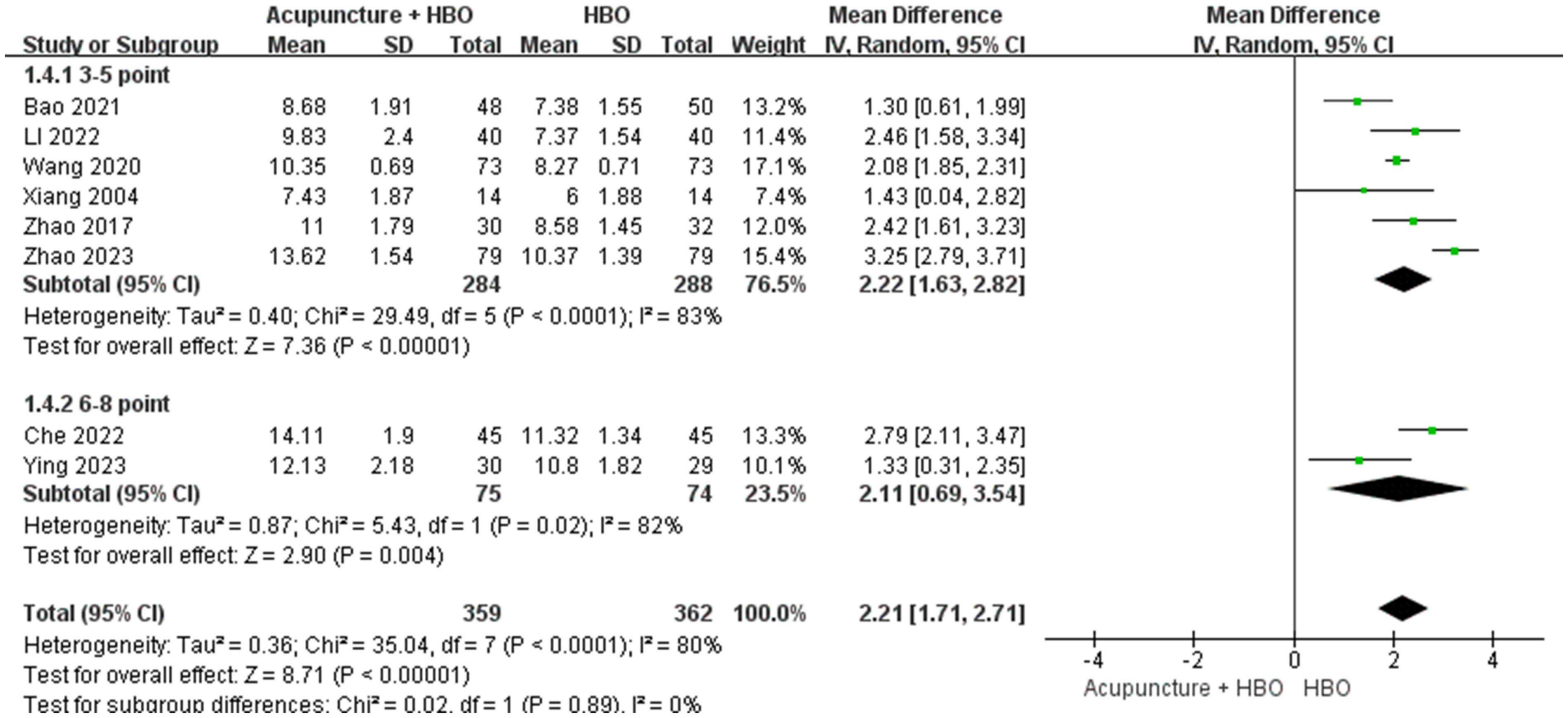
Forest plot of effects of acupuncture combined with HBO versus HBO alone on GCS score (Subgroup by the different varying degrees of severe TBI). MD > 0 indicates that the acupuncture combined with HBO group demonstrated higher GCS scores.

Subgroup analysis accounting for baseline injury severity revealed critical methodological considerations. The heterogeneity in baseline GCS scores constituted a potential confounding factor, given established evidence of a graded association between initial GCS scores and neurological prognosis in TBI patients (30). As none of the included studies implemented GCS-stratified analysis or covariate adjustment, we conducted *post hoc* subgroup analyses. After excluding a single-centre study (24) enrolling patients with GCS > 8, the pooled effect size remained robust (MD = 2.21, 95% CI: 1.71–2.71) (Figure 6). The remaining eight studies focusing on severe TBI (GCS ≤ 8) were stratified using a cutoff of GCS score: very severe subgroup (GCS 3–5, *n* = 6) and severe subgroup (GCS 6–8, *n* = 2). Random-effects model synthesis demonstrated comparable efficacy between subgroups (test for subgroup differences: *p* = 0.27), though point estimates suggested marginally more significant improvement in the very severe subgroup (MD = 2.22, 95% CI: 1.63–2.82, *p* < 0.001) compared with the severe subgroup (MD = 2.11, 95% CI: 0.69–3.54, *p* = 0.004) (Figure 7).

**Figure 8:**
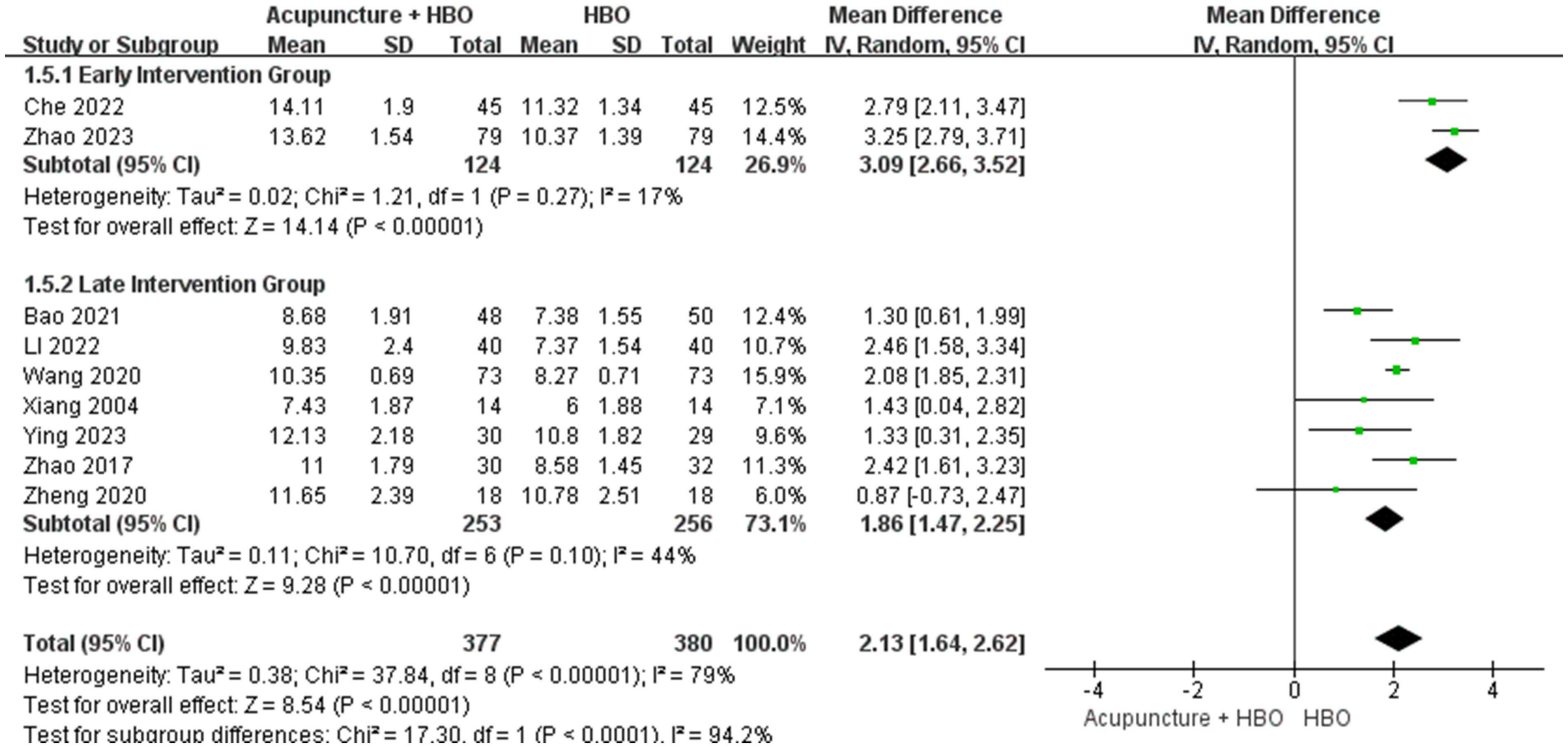
Forest plot of effects of acupuncture combined with HBO versus HBO alone on GCS score (Subgroup by the different acupoint combination). MD > 0 indicates that the acupuncture combined with HBO group demonstrated higher GCS scores.

Notably, temporal heterogeneity remained unresolved. Although the 7- to 14-day post-injury window has been proposed as a critical period for neurological recovery in severe TBI (31), significant variability existed in time-to-intervention across original studies. To address this, we stratified the nine studies into early (≤14 days post-injury, *n* = 2) and late (>14 days, *n* = 7) intervention subgroups. Random-effects meta-analysis revealed substantial between-subgroup heterogeneity (*p* < 0.00001, I^2^ = 92.6%), with the early intervention subgroup demonstrating superior GCS improvement (MD = 3.09, 95% CI: 2.66–3.52) compared to the late subgroup (MD = 1.86, 95% CI: 1.47–2.25) (Figure 8).

#### 3.5.2 Consciousness rate

**Figure 9:**
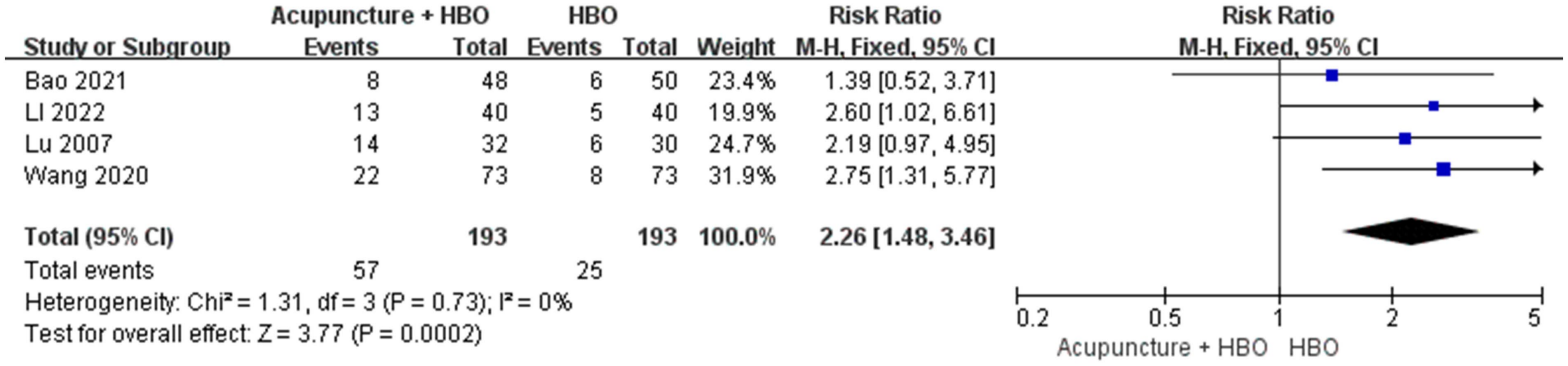
Forest plot of effects of acupuncture combined with HBO versus HBO alone on Consciousness rate. RR > 1 indicates that the acupuncture combined with HBO group demonstrated higher CR.

Four studies implementing acupuncture combined with HBO reported the CR (19, 21, 26, 29). Among the 193 patients included in the control groups in these studies, 25 were awake after treatment, resulting in a CR of 12.95%. Among the 193 patients included in the test groups, 57 were awake, resulting in a CR of 29.53%. There was no significant heterogeneity among the studies (*p* = 0.73, I^2^ = 0). The results indicated a substantial difference in the post-treatment CR between the two groups (RR = 2.26, 95% CI: 1.48 to 3.46, *p* = 0.0002) (Figure 9).

#### 3.5.3 Effective rate

**Figure 10:**
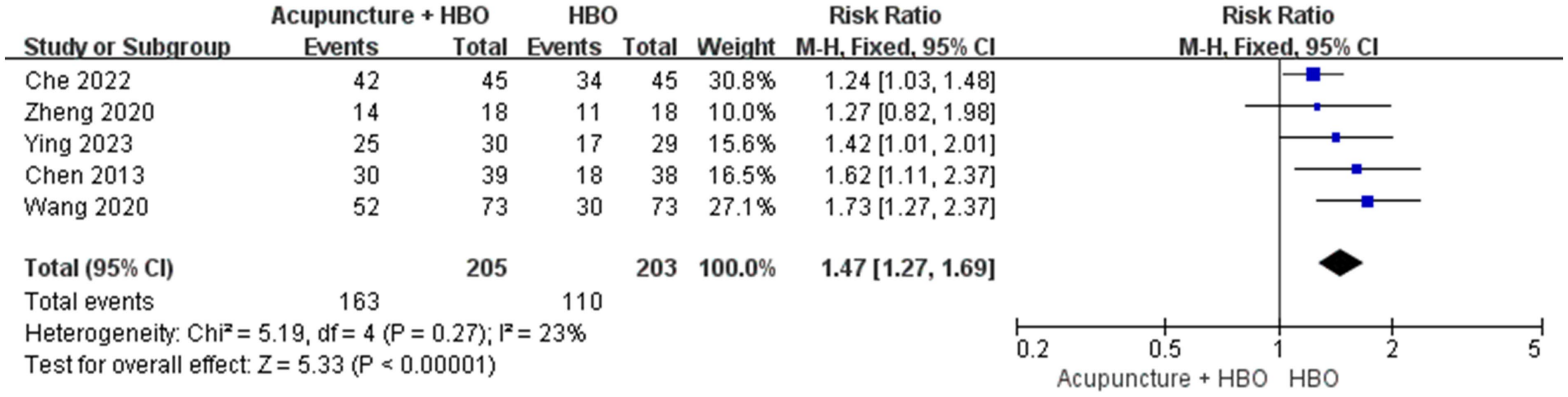
Forest plot of effects of acupuncture combined with hyperbaric oxygenation versus hyperbaric oxygenation alone on Effective rate. RR > 1 indicates that the acupuncture combined with HBO group demonstrated higher ER.

Four studies reported the ER (20, 24, 26–28). A total of 203 individuals were enrolled in the control group, while 110 patients exhibited a favourable prognosis for an ER of 54.19%. A total of 205 individuals were enrolled in the control group, while 163 patients exhibited a favourable prognosis for an ER of 79.51%. No significant heterogeneity was observed among the studies (*p* = 0.27, I^2^ = 23). Consequently, a fixed-effects model was employed in the meta-analysis. The results indicated that there was a significant difference in the RR between the two groups (RR = 1.47, 95% CI: 1.27 to 1.69, *p* < 0.00001) (Figure 10).

### 3.6 Side effects and adverse events

Only one study (23) reported that some patients who received acupuncture treatment experienced pain and fever, though there was no attrition.

### 3.7 Publication bias

**Figure 11:**
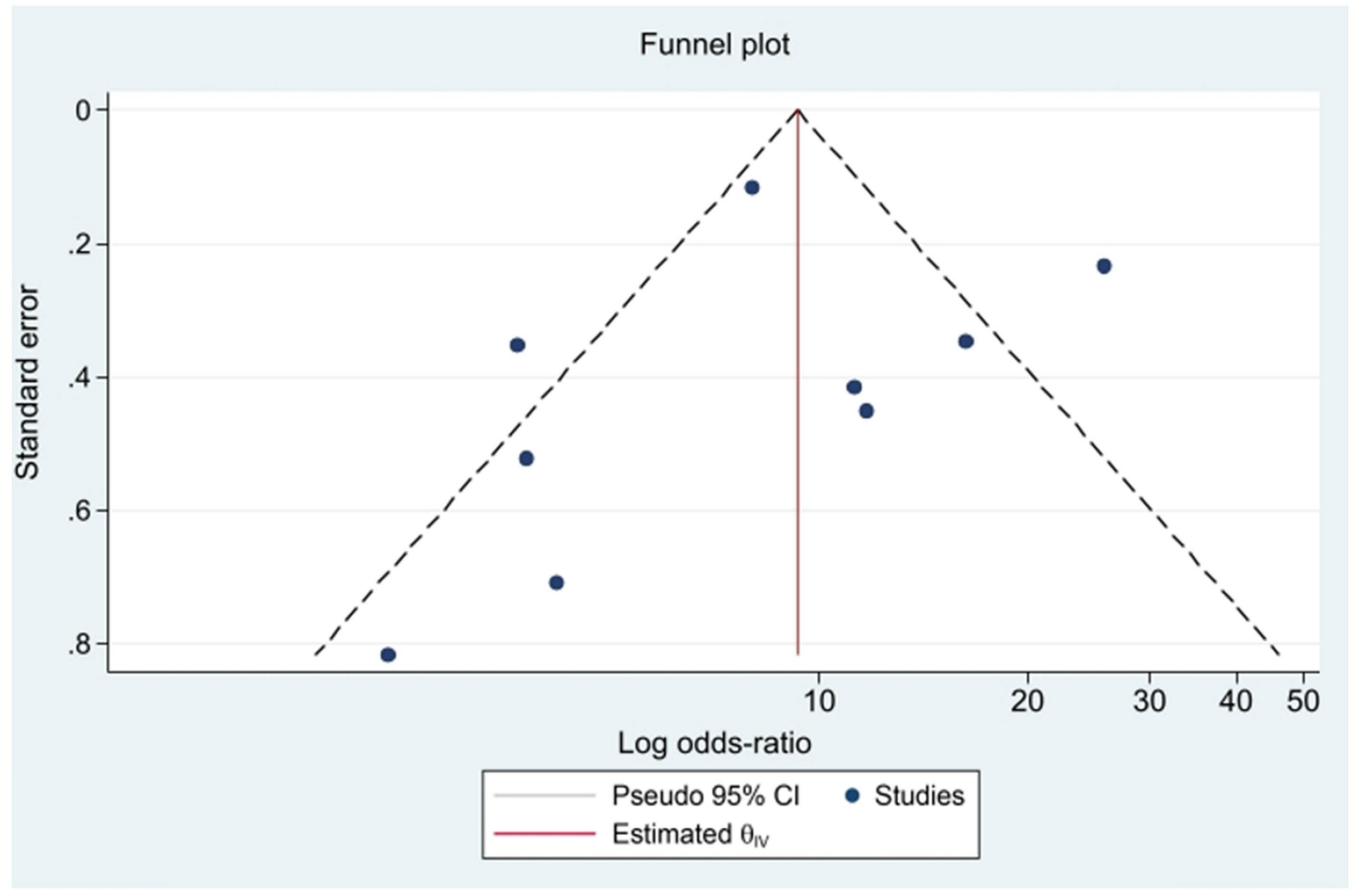
Funnel plot for publication bias detection.

When the number of samples included in the GCS score change exceeded 8, an inverted funnel plot was drawn. Figure 11 shows the funnel plot, which appears symmetrical. Egger’s regression test and Begg’s funnel plot asymmetry test yielded *p* values > 0.05 for all tests, indicating the absence of significant publication bias.

## 4 Discussion

This study presents the first systematic meta-analysis evaluating the synergistic effects of acupuncture combined with HBO in TBI. Pooled data from 11 randomized controlled trials (*n* = 896 patients) demonstrated significantly more significant improvement in GCS scores with combination therapy (MD = 2.13, 95% CI: 1.64–2.62, *p* < 0.001), with the EA subgroup exhibiting the highest effect size (MD = 2.15, 95% CI: 1.95–2.36). These findings provide critical evidence for clinical translation, suggesting that EA-HBO combination therapy may overcome therapeutic resistance in severe TBI patients (GCS ≤ 8) refractory to conventional treatments.

The superior efficacy of EA may stem from its dual neuromodulatory mechanisms: (1). Electrical pulse stimulation enhances acupoint-mediated regulation of central neural circuits, potentially through increased neurotrophic factor release (e.g., BDNF) and cerebral blood flow optimization (32); (2). Direct current stimulation may reactivate residual neuronal networks in lesioned areas (33), creating spatiotemporal synergy with HBO’s neurorestorative effects through oxygen metabolism potentiation.

Subgroup analyses further elucidated critical effect modifiers: baseline injury severity and therapeutic timing. The very severe TBI subgroup (GCS 3–5) exhibited greater precision (CI *Δ* = 1.19 vs. 2.85), potentially attributable to lower therapeutic response thresholds (e.g., pupillary reflex recovery conferring disproportionate GCS gains), whereas the severe subgroup (GCS 6–8) required higher-order functional recovery (e.g., verbal response). Early intervention demonstrated superior efficacy (MD = 3.09 vs. 1.86), suggesting temporally stratified biological mechanisms: (1). Acute phase (≤14 days): Elevated blood–brain barrier permeability (34) enhances HBO-mediated oxygen diffusion, while acupuncture potentiates synaptic remodelling (35); (2). Microglial phenotypic shift from pro-inflammatory M1 to reparative M2 dominates (36), fostering anti-inflammatory niches; (3). Chronic phase (>14 days): Astroglial scar formation increases extracellular matrix stiffness, biomechanically impeding therapeutic agent delivery (37).

The conclusions of this study align with prior evidence while advancing the field methodologically. A 2018 meta-analysis (38) confirmed acupuncture’s efficacy in improving TBI-related consciousness disorders (RR = 1.58), but critical limitations persist: it neither stratified outcomes by acupuncture modality nor assessed combination therapies, and included studies only up to 2018, limiting its applicability to contemporary multimodal therapeutic strategies.

Having established the clinical superiority of combination therapy, elucidating its underlying synergistic mechanisms becomes pivotal for guiding precision treatment. Although numerous studies have proposed potential mechanisms of acupuncture or HBO monotherapy in TBI, research explicitly investigating the synergistic mechanisms of their combined application remains limited. Based on existing evidence, we hypothesize that the synergistic mechanisms of combined acupuncture-HBO therapy may involve the following dimensions: (1) HBO therapy enhances cerebral oxygen supply and optimizes metabolic homeostasis (39), thereby establishing an oxygen-enriched microenvironment that potentiates acupuncture-mediated neurotransmitter regulation and metabolic modulation (40); (2). The combined therapy of acupuncture and HBO exerts multi-pathway, multitarget anti-inflammatory effects, effectively mitigating cerebral tissue damage (41–43); (3) Both modalities enhance systemic antioxidant capacity, and their combined use synergistically augments free radical scavenging efficacy, thereby mitigating oxidative damage to cerebral tissue (44, 45). (4) The combination therapy promotes neuroplasticity through complementary mechanisms: HBO establishes an oxygen-repleted microenvironment post-hypoxia (46), while acupuncture directly enhances neuronal excitability and synaptic plasticity (47). (5) The combined therapy exerts neuroprotective effects by suppressing neuronal injury and apoptosis through distinct molecular pathways (48, 49). In summary, the synergistic mechanisms underlying the combined treatment of acupuncture and hyperbaric oxygen (HBO) therapy for traumatic brain injury (TBI) exhibit multi-dimensional and multitarget characteristics. However, these current hypotheses are predominantly based on experimental studies investigating their individual effects. Future research necessitates additional experimental studies and clinical trials to validate further and clarify the synergistic mechanisms of this combined therapeutic approach.

However, the reliability of evidence in this study is constrained by methodological limitations. Three studies (22, 26, 28) failed to describe randomization details, and all included studies lacked allocation concealment and blinding, resulting in elevated risks of selection bias and detection bias. Although subgroup analyses demonstrated significant GCS score improvement with manual acupuncture combined with HBO (MD = 1.59, 95% CI: 1.12–2.05) and superior consciousness recovery rates with the combination therapy (RR = 2.26, 95% CI:1.48–3.46), the wide confidence intervals indicate residual uncertainty, which likely stems from small-sample effects (only four studies were included across analyses, minimum sample size *n* = 36) and rare-event bias (limited awakening cases post-combination therapy in comatose TBI patients). The future studies must rigorously adhere to CONSORT guidelines by conducting large-scale RCTs with standardized interventions, explicitly reporting stimulation parameters, and implementing enhanced methodologies: double-blinding protocols, blockchain-based centralized randomization systems for real-time encrypted allocation sequencing, and auditable allocation processes with tamper-proof documentation.

## 5 Conclusion

In conclusion, our meta-analysis results indicate that the combination of acupuncture with HBO exerts a positive influence on GCS scores, CR, and ER in patients with TBI. However, given the limited availability of high-quality evidence and the dearth of RCTs in this area, our ability to definitively evaluate the comparative efficacy of acupuncture in conjunction with HBO versus HBO monotherapy for TBI treatment is compromised.

## Data Availability

The original contributions presented in the study are included in the article/Supplementary material, further inquiries can be directed to the corresponding author.
