# Supplementary material for: The effect of acupuncture combined with hyperbaric oxygenation compared with hyperbaric oxygenation alone for patients with traumatic brain injury: a systematic review and meta-analysis
Source: Front Neurol. 2025 May 2;16:1538740. doi: 10.3389/fneur.2025.1538740 (PMC12083083; doi:10.3389/fneur.2025.1538740)
Supplement: Supplementary file 1 [file Data_Sheet_1.docx]

**CNKI：**

( TI % ' craniocerebral trauma ' OR TI % ' brain trauma ' OR TI % ' traumatic brain injury ' OR TI % ' craniocerebral injury ' ) AND (SU % ' acupuncture ' OR SU % ' electroacupuncture ' OR SU % ' fire needle ' OR SU % ' hand acupuncture ' OR SU % ' body acupuncture ' OR SU % ' scalp acupuncture ' OR SU % ' wrist-ankle acupuncture ' OR SU % ' abdominal acupuncture ' OR SU % ' mild moxibustion ' OR SU % ' acupoint catgut embedding ' OR SU % ' moxibustion ' OR SU % ' warm acupuncture ' OR SU % ' thunder-fire moxibustion ' OR SU % ' grain-sized moxibustion ') AND ( SU % ' Hyperbaric Oxygenation ' )

108

**万方**：

Title: (craniocerebral trauma OR brain trauma OR traumatic brain injury OR craniocerebral injury) and Theme: (acupuncture OR electroacupuncture OR fire needle OR hand acupuncture OR body acupuncture OR scalp acupuncture OR wrist-ankle acupuncture OR abdominal acupuncture OR acupoint catgut embedding OR mild moxibustion OR moxibustion OR warm acupuncture OR thunder-fire moxibustion OR grain-sized moxibustion) and Theme: (Hyperbaric Oxygenation)

95

**维普：**

M = (craniocerebral trauma OR brain trauma OR traumatic brain injury OR craniocerebral injury) AND R = (acupuncture OR electroacupuncture OR fire needle OR hand acupuncture OR body acupuncture OR scalp acupuncture OR wrist-ankle acupuncture OR abdominal acupuncture OR acupoint catgut embedding OR mild moxibustion OR moxibustion OR warm acupuncture OR thunder-fire moxibustion OR grain-sized moxibustion) AND R = (Hyperbaric Oxygenation)

65

**Sinomed：**

[( " craniocerebral trauma "[ Common fields: Intelligence] OR " brain trauma "[ Common fields: Intelligence] OR " traumatic brain injury "[ Common fields: Intelligence] OR " craniocerebral injury "[ Common fields: Intelligence]) AND( " acupuncture "[ Common fields: Intelligence] OR " electroacupuncture " [ Common fields: Intelligence] OR " fire needle "[ Common fields: Intelligence] OR " hand acupuncture "[ Common fields: Intelligence] OR " body acupuncture "[ Common fields: Intelligence] OR " scalp acupuncture "[ Common fields: Intelligence] OR " wrist-ankle acupuncture "[ Common fields: Intelligence] OR " abdominal acupuncture "[ Common fields: Intelligence] OR " acupoint catgut embedding "[ Common fields: Intelligence] OR " mild moxibustion OR “ moxibustion ”[ Common fields: Intelligence] OR " warm acupuncture "[ Common fields: Intelligence] OR " thunder-fire moxibustion "[ Common fields: Intelligence] OR " grain-sized moxibustion "[ Common fields: Intelligence]) AND " Hyperbaric Oxygenation "[ Common fields: Intelligence]](javascript:toDoRelimitSearch();)

89

**PubMed：**

#1 "brain injuries, traumatic"[MeSH Terms] 27379

#2 ((((((aumatic brain injury [Title/Abstract]) OR (brain injury [Title/Abstract])) OR (TBI[Title/Abstract])) OR (craniocerebral trauma [Title/Abstract])) OR (craniocerebral injury [Title/Abstract])) OR (head injury [Title/Abstract])) OR (head trauma [Title/Abstract]) 119482

#3 #1 OR #2

#4 "Acupuncture"[MeSH Terms] 2098

#5 (((((((((((electroacupuncture[Title/Abstract]) OR (fire acupuncture[Title/Abstract])) OR (hand acupuncture[Title/Abstract])) OR (body acupuncture[Title/Abstract])) OR (head acupuncture[Title/Abstract])) OR (wrist ankle acupuncture[Title/Abstract])) OR (abdominal acupuncture[Title/Abstract])) OR (acupoint thread embedding[Title/Abstract])) OR (moxibustion[Title/Abstract])) OR (mild moxibustion[Title/Abstract])) OR (warm acupuncture[Title/Abstract])) OR (thunder fire moxibustion[Title/Abstract])) OR (wheat grain moxibustion[Title/Abstract]) 11488

#6 #4 OR #5 13086

#7 #3 AND #6 121

#8 Hyperbaric Oxygenation"[Mesh] 12922

#9 hyperbaric chamber [Title/Abstract] 851

#10 #8 OR #9 13318

#11 #7 AND #10 0

**Cochrane library**

#1 MeSH descriptor: [Brain Injuries, Traumatic] explode all trees 1691

#2 ("aumatic brain injury"):ti,ab,kw OR ("brain injury"):ti,ab,kw OR (TBI):ti,ab,kw OR ("craniocerebral trauma"):ti,ab,kw OR ("craniocerebral injury"):ti,ab,kw 9062

#3 ("head injury"):ti,ab,kw OR ("head trauma"):ti,ab,kw 2160

#4 #1 OR #2 OR #3 10585

#5 MeSH descriptor: [Acupuncture] explode all trees 224

#6 (electroacupuncture):ti,ab,kw OR ("fire acupuncture"):ti,ab,kw OR ("hand acupuncture"):ti,ab,kw OR ("body acupuncture"):ti,ab,kw OR ("head acupuncture"):ti,ab,kw 3976

#7 ("wrist ankle acupuncture"):ti,ab,kw OR ("abdominal acupuncture"):ti,ab,kw OR ("acupoint thread embedding"):ti,ab,kw OR ("mild moxibustion"):ti,ab,kw OR ("warm acupuncture"):ti,ab,kw 424

#8 ("thunder fire moxibustion"):ti,ab,kw OR ("wheat grain moxibustion"):ti,ab,kw OR (moxibustion):ti,ab,kw 2603

#9 #5 OR #6 OR #7 OR #8 6859

#10 #4 AND #9 37

#11 MeSH descriptor: [Hyperbaric Oxygenation] explode all trees 616

#12 #10 AND #11

n=0

**Embase**

'traumatic brain injury'/exp OR 'aumatic brain injury':ti,ab,kw OR 'brain injury':ti,ab,kw OR 'craniocerebral trauma':ti,ab,kw OR 'craniocerebral injury':ti,ab,kw OR 'head injury':ti,ab,kw OR 'head trauma':ti,ab,kw) AND ('acupuncture'/exp OR electroacupuncture:ti,ab,kw OR 'fire acupuncture':ti,ab,kw OR 'head acupuncture':ti,ab,kw OR 'hand acupuncture':ti,ab,kw OR 'body acupuncture':ti,ab,kw OR 'wrist ankle acupuncture':ti,ab,kw OR 'abdominal acupuncture':ti,ab,kw OR 'acupoint thread embedding':ti,ab,kw OR moxibustion:ti,ab,kw OR 'warm acupuncture':ti,ab,kw OR 'thunder fire moxibustion':ti,ab,kw OR 'wheat grain moxibustion':ti,ab,kw) AND ('hyperbaric oxygen therapy'/exp OR 'hyperbaric chamber':ti,ab,kw)

n=23

**Web of science**

#1 TS=("brain injuries, traumatic") OR TS=("aumatic brain injury") OR TS=("brain injury") OR TS=(TBI) OR TS=("craniocerebral trauma") OR TS=("head acupuncture") OR TS=("craniocerebral injury") OR TS=("head injury") OR TS=("head trauma")

#2 TS=("Acupuncture") OR TS=("electroacupuncture") OR TS=("fire acupuncture") OR TS=("hand acupuncture") OR TS=("body acupuncture") OR TS=("head acupuncture") OR TS=("wrist ankle acupuncture") OR TS=("abdominal acupuncture") OR TS=("acupoint thread embedding") OR TS=("moxibustion") OR TS=("mild moxibustion") OR TS=("warm acupuncture") OR TS=("thunder fire moxibustion") OR TS=("wheat grain moxibustion")

#3 (TS= Hyperbaric Oxygenation)

#4 #1 AND #2 AND #3

N=2
